# Supplementary material for: Chondromodulin is necessary for cartilage callus distraction in mice
Source: PLoS One. 2023 Feb 16;18(2):e0280634. doi: 10.1371/journal.pone.0280634 (PMC9934371; doi:10.1371/journal.pone.0280634)
Supplement: S1 Table — (DOCX) [file pone.0280634.s001.docx]

| **Supplemental Table 1.** Pre-validated Assays on Demand™ (mix of unlabelled PCR primers and Taq-Man^®^ MGB probe (FAM™ dye labelled)) | |
| --- | --- |
| Gene | Catalogue Number |
| ***Cnmd*** | Mm00504347_m1 |
| ***Sox9*** | Mm00448840_m1 |
| ***Col2a1*** | Mm00491889_m1 |
| ***Col10a1*** | Mm00487041_m1 |
| ***Vegf*** | Mm01281449_m1 |
| ***Mmp2*** | Mm00439498_m1 |
| ***Mmp9*** | Mm00442991_m1 |
| ***Mmp14*** | Mm00485054_m1 |
| ***Tnmd*** | Mm00491594_m1 |
| ***Timp2*** | Mm00441825_m1 |
| ***Gapdh*** | Mm99999915_g1 |
