# Supplementary figures and images for: Chondromodulin is necessary for cartilage callus distraction in mice
Source: PLoS One. 2023 Feb 16;18(2):e0280634. doi: 10.1371/journal.pone.0280634 (PMC9934371; doi:10.1371/journal.pone.0280634)

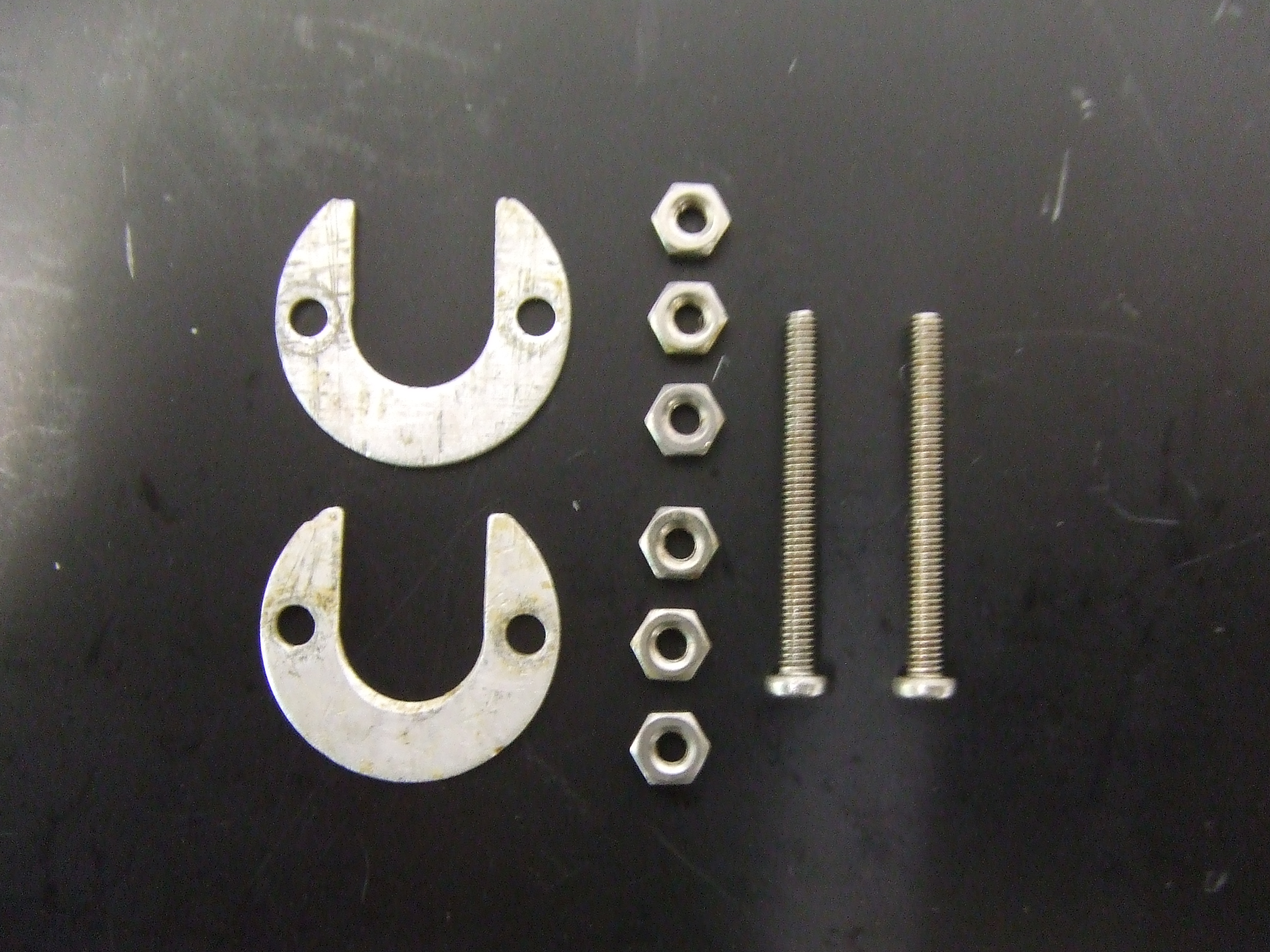

Supplement: S1 File — (ZIP) [file pone.0280634.s002.zip › Yamaguchi University/Figure 1/External fixator1.JPG]

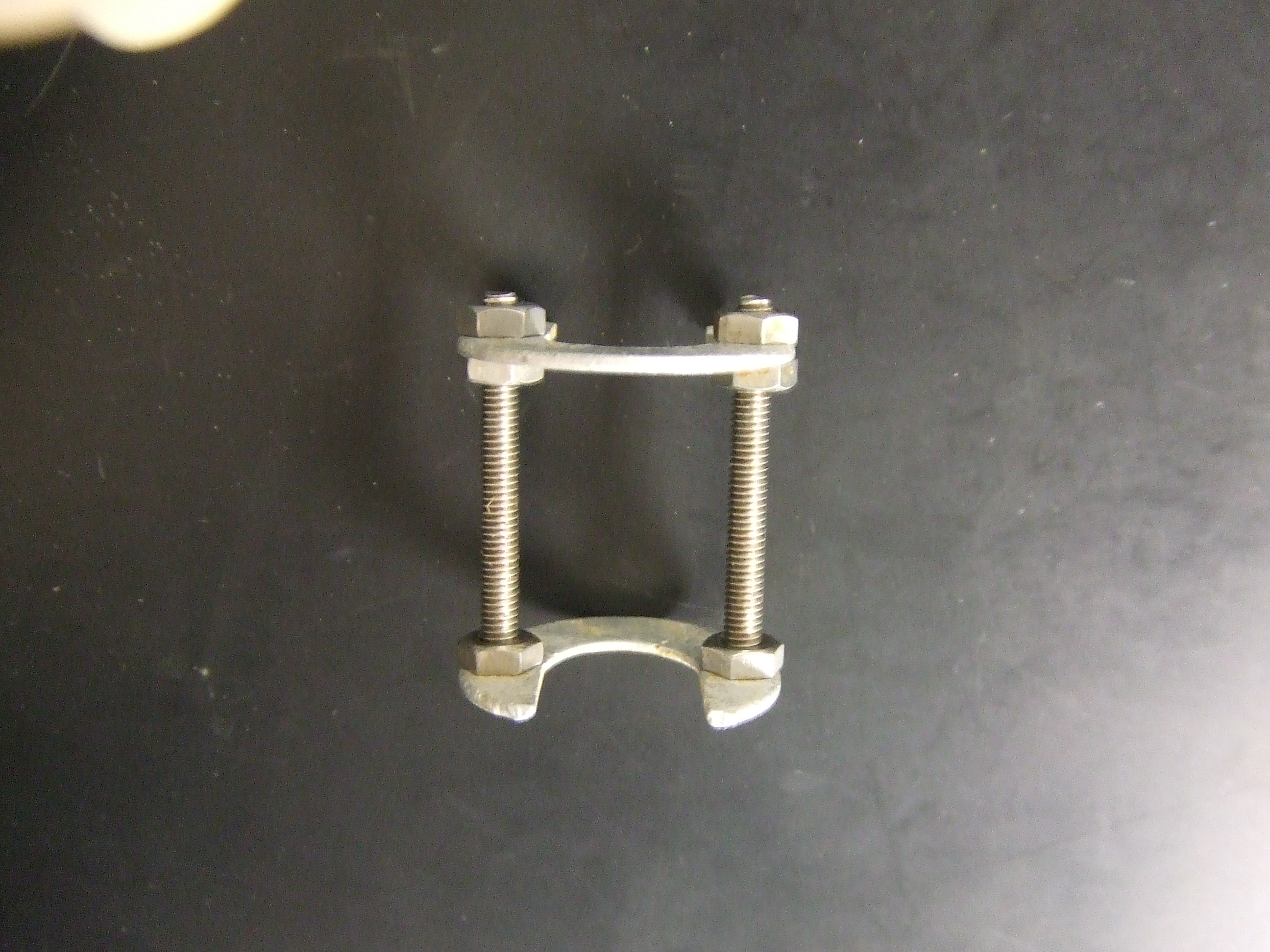

Supplement: S1 File — (ZIP) [file pone.0280634.s002.zip › Yamaguchi University/Figure 1/External fixator2.JPG]

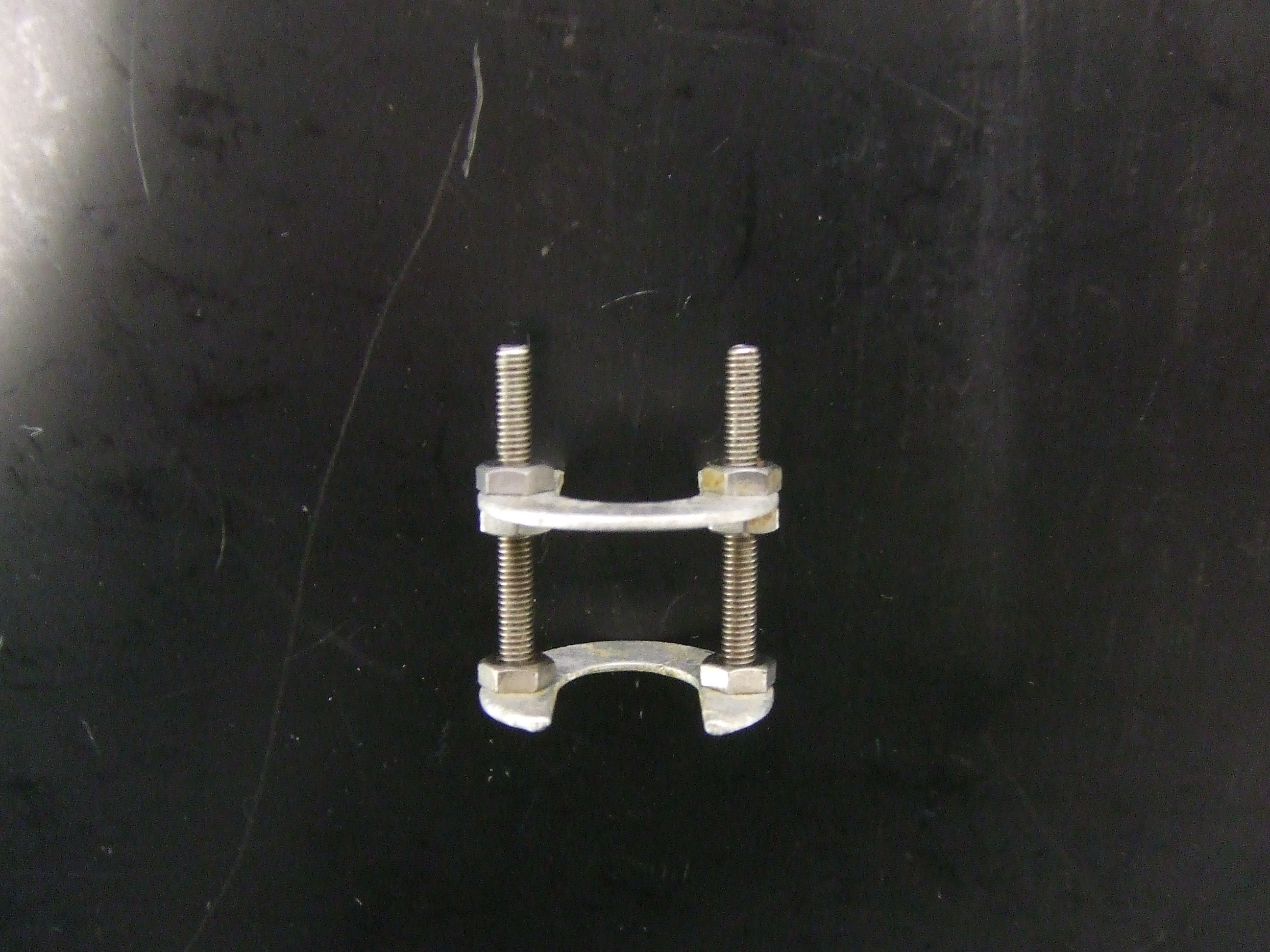

Supplement: S1 File — (ZIP) [file pone.0280634.s002.zip › Yamaguchi University/Figure 1/External fixator3.JPG]

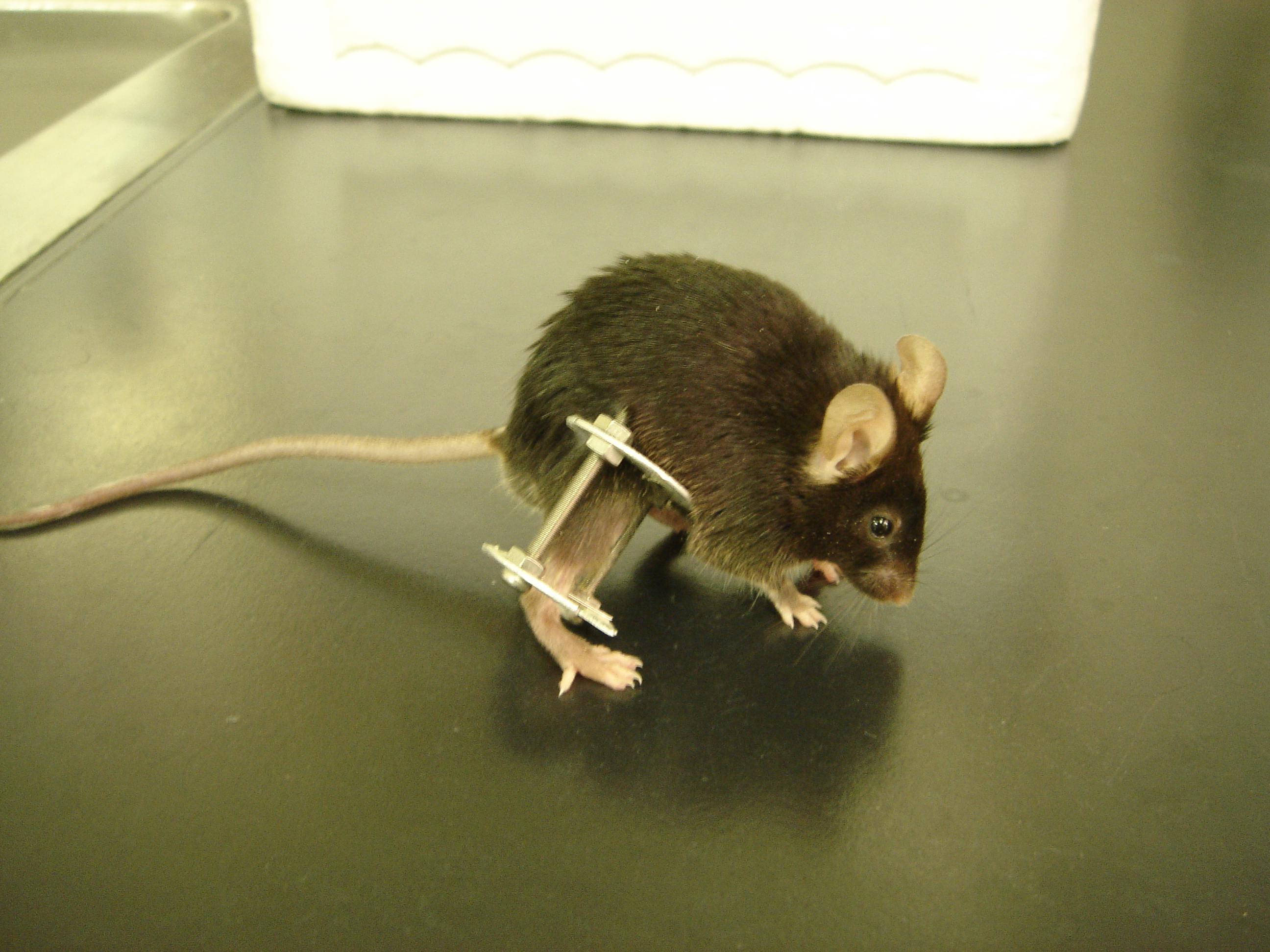

Supplement: S1 File — (ZIP) [file pone.0280634.s002.zip › Yamaguchi University/Figure 1/mouse with EF.JPG]

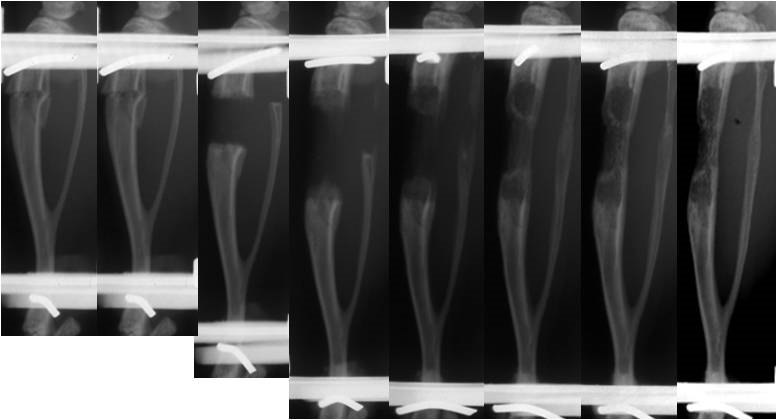

Supplement: S1 File — (ZIP) [file pone.0280634.s002.zip › Yamaguchi University/Figure 2/Cnmd KO X-P.tif]

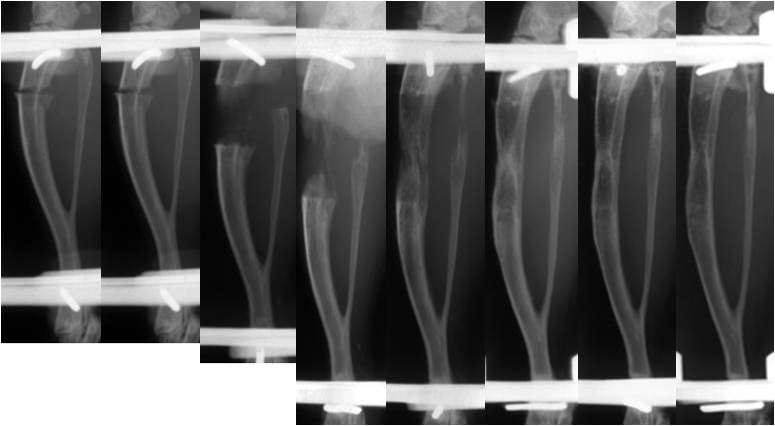

Supplement: S1 File — (ZIP) [file pone.0280634.s002.zip › Yamaguchi University/Figure 2/Wild-type X-P.tif]

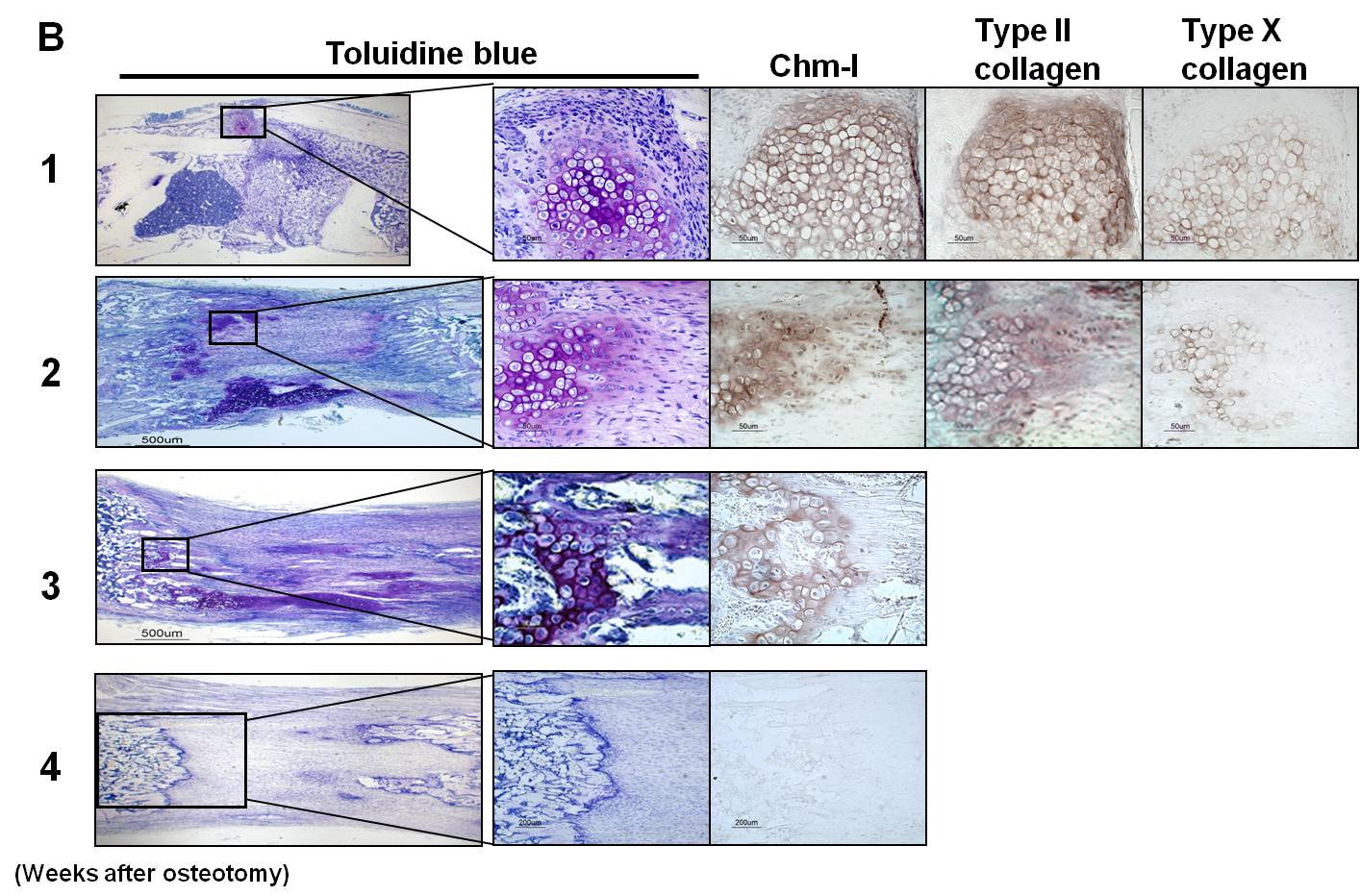

Supplement: S1 File — (ZIP) [file pone.0280634.s002.zip › Yamaguchi University/Figure 3/immunostaining.jpg]

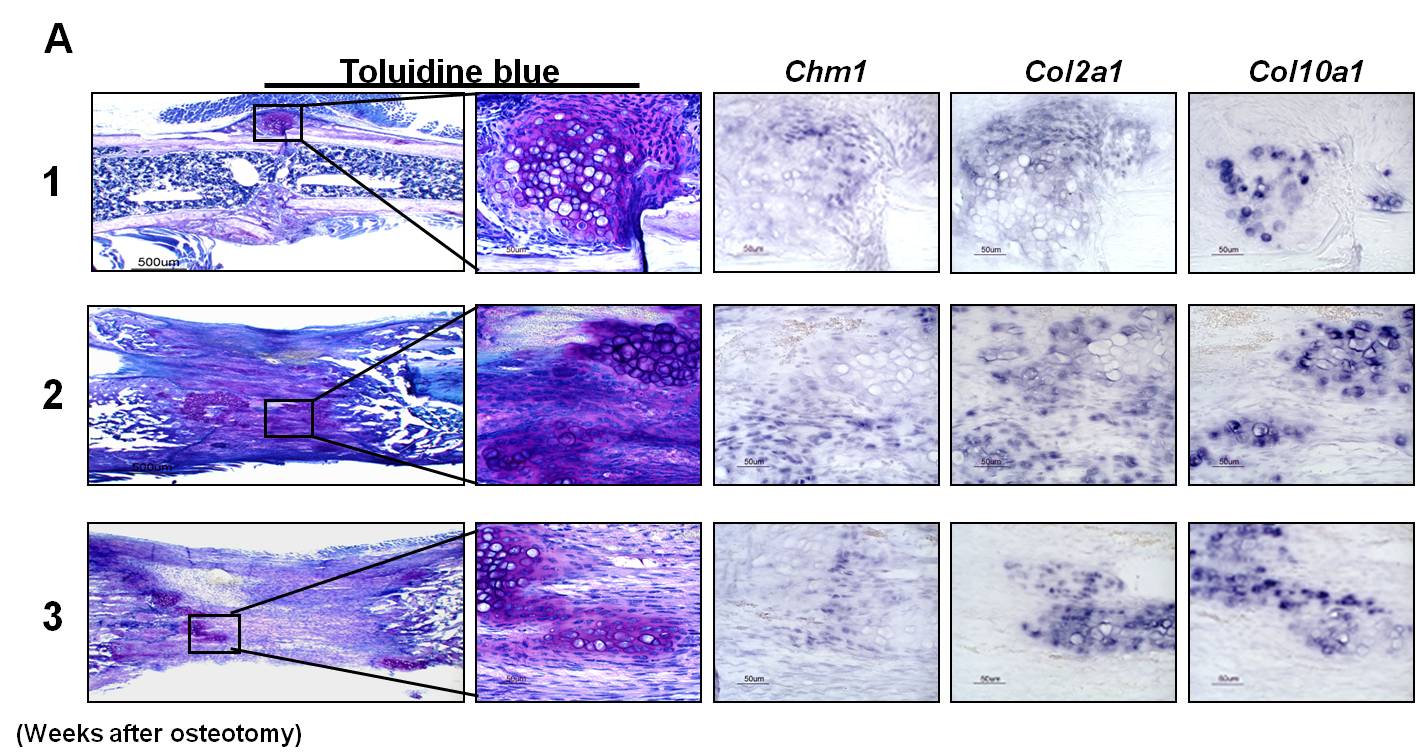

Supplement: S1 File — (ZIP) [file pone.0280634.s002.zip › Yamaguchi University/Figure 3/ISH.jpg]

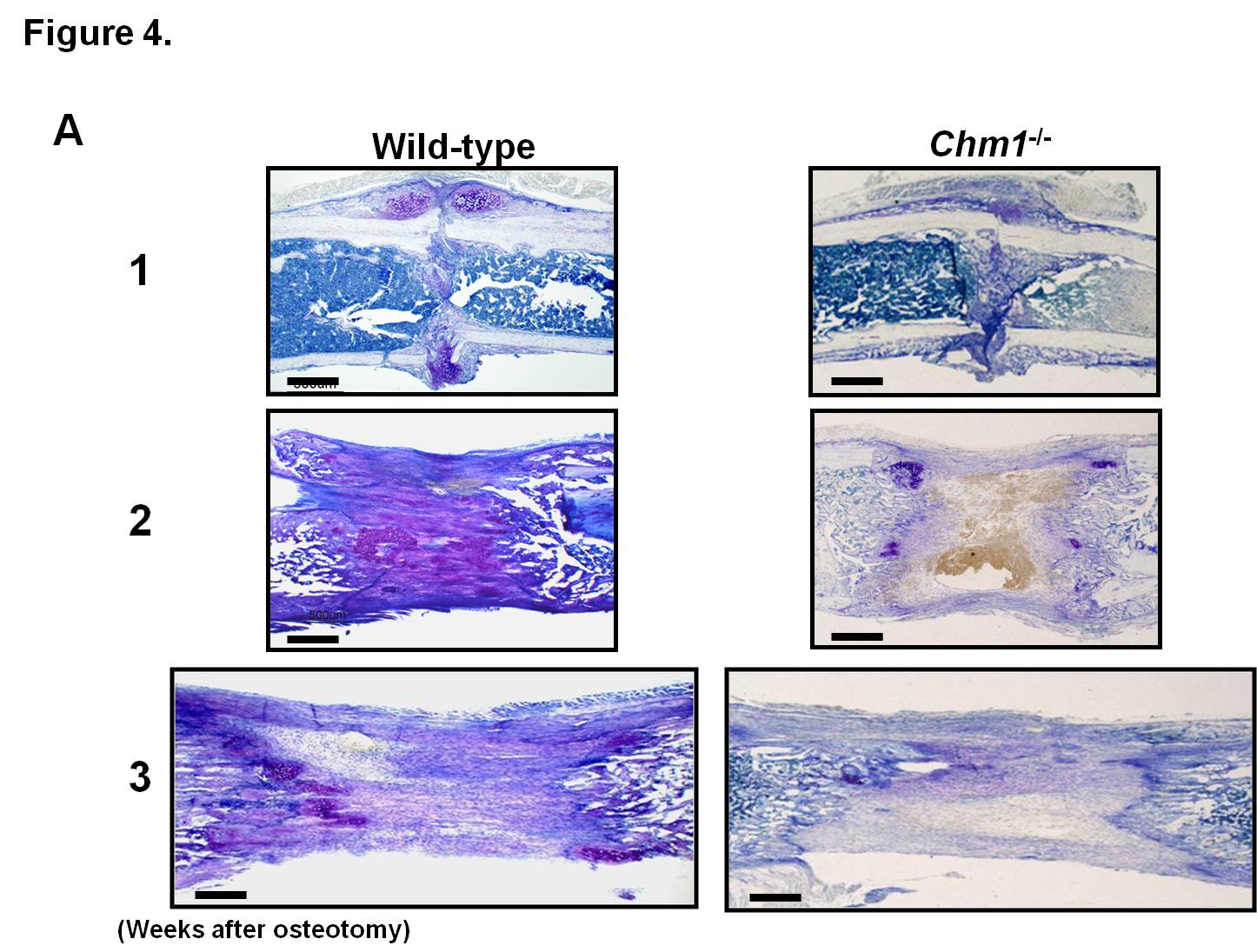

Supplement: S1 File — (ZIP) [file pone.0280634.s002.zip › Yamaguchi University/Figure 4/fig 4.jpg]

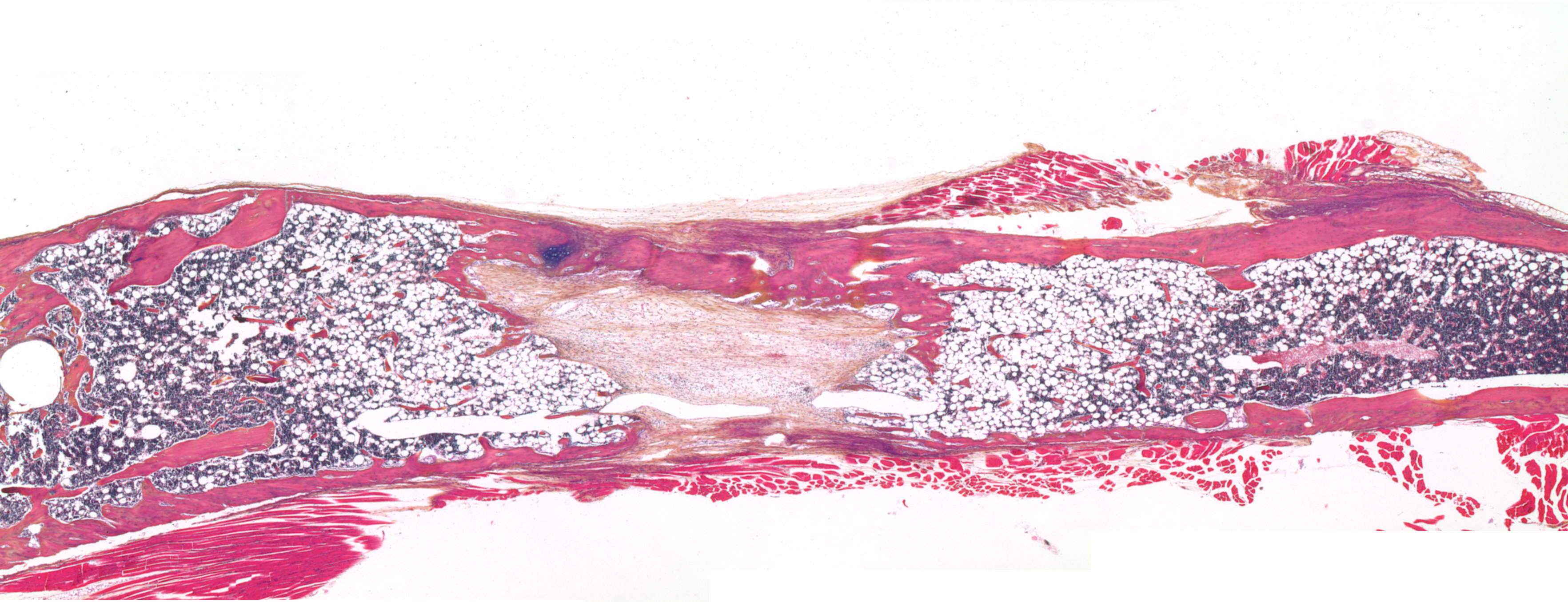

Supplement: S1 File — (ZIP) [file pone.0280634.s002.zip › Yamaguchi University/Figure 5/HE staining/Cnmd KO 12w(2).jpg]

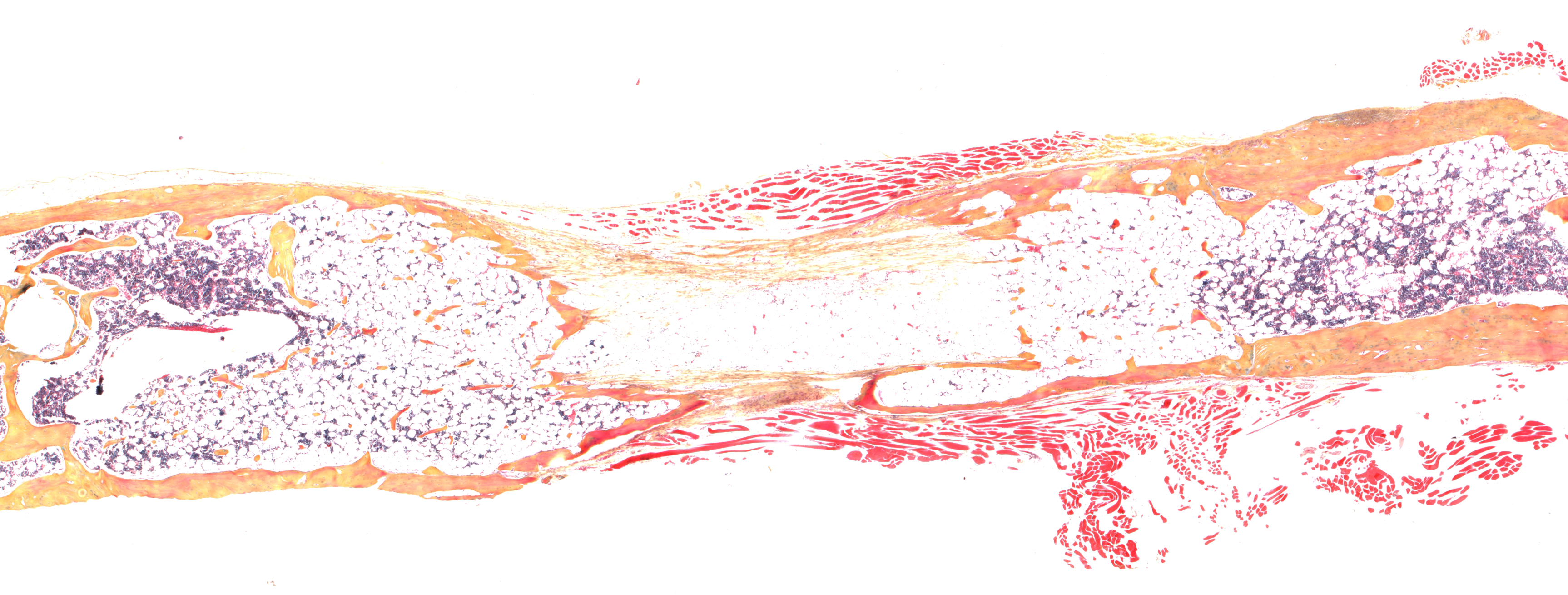

Supplement: S1 File — (ZIP) [file pone.0280634.s002.zip › Yamaguchi University/Figure 5/HE staining/Cnmd KO 12w(3).jpg]

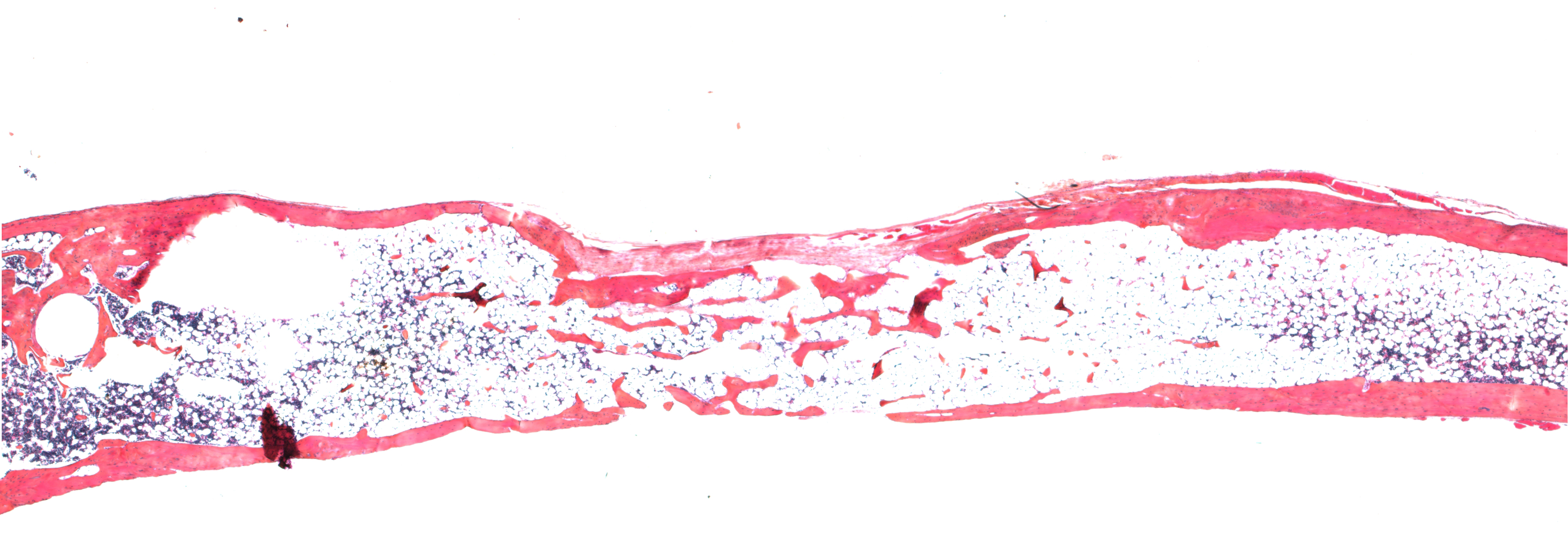

Supplement: S1 File — (ZIP) [file pone.0280634.s002.zip › Yamaguchi University/Figure 5/HE staining/Cnmd KO 12w.jpg]

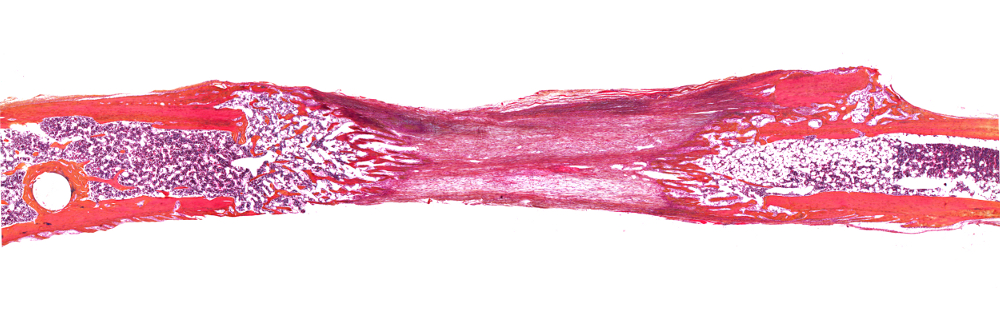

Supplement: S1 File — (ZIP) [file pone.0280634.s002.zip › Yamaguchi University/Figure 5/HE staining/Cnmd KO 3w.jpg]

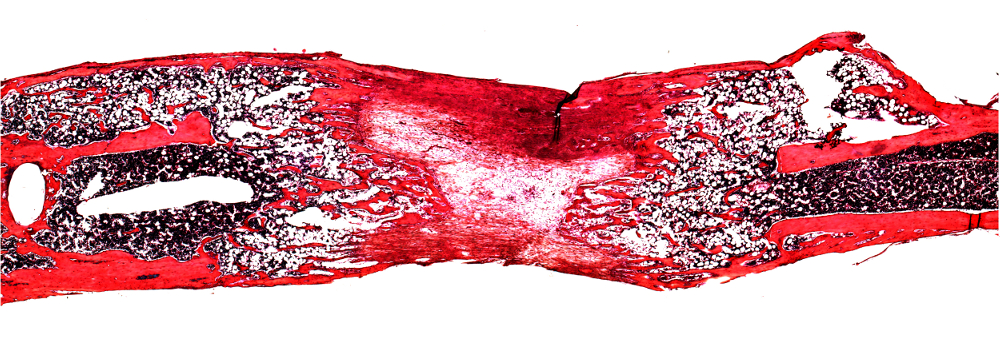

Supplement: S1 File — (ZIP) [file pone.0280634.s002.zip › Yamaguchi University/Figure 5/HE staining/Cnmd KO 4w.jpg]

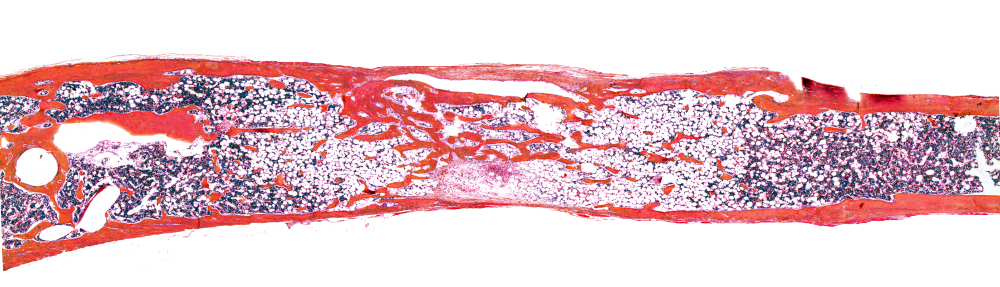

Supplement: S1 File — (ZIP) [file pone.0280634.s002.zip › Yamaguchi University/Figure 5/HE staining/Cnmd KO 6w.jpg]

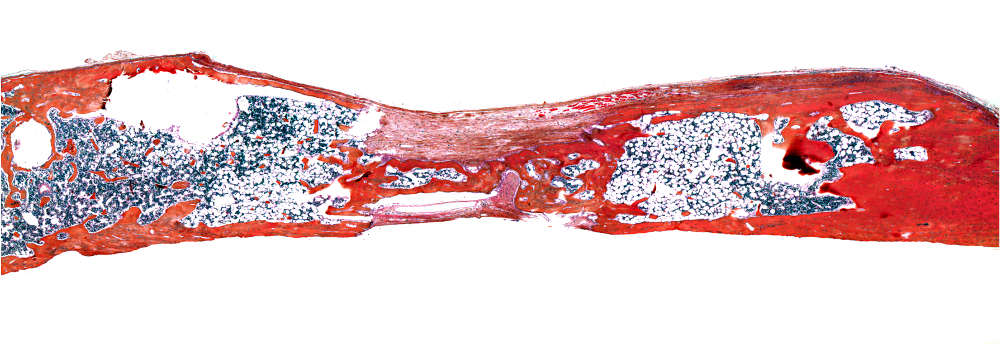

Supplement: S1 File — (ZIP) [file pone.0280634.s002.zip › Yamaguchi University/Figure 5/HE staining/Cnmd KO 8w.jpg]

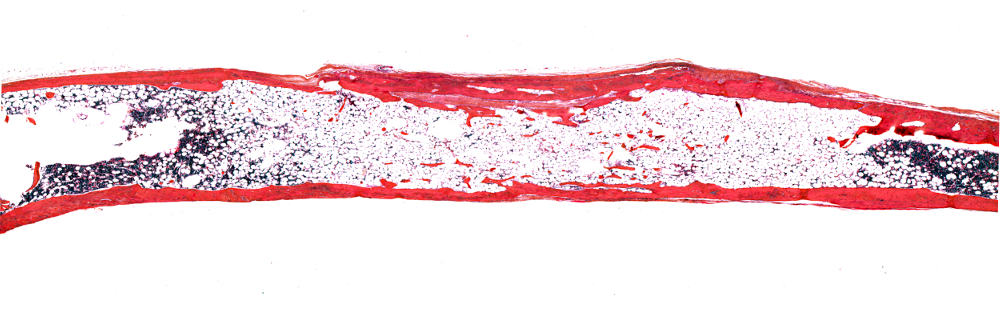

Supplement: S1 File — (ZIP) [file pone.0280634.s002.zip › Yamaguchi University/Figure 5/HE staining/Wild-type 12w (1).jpg]

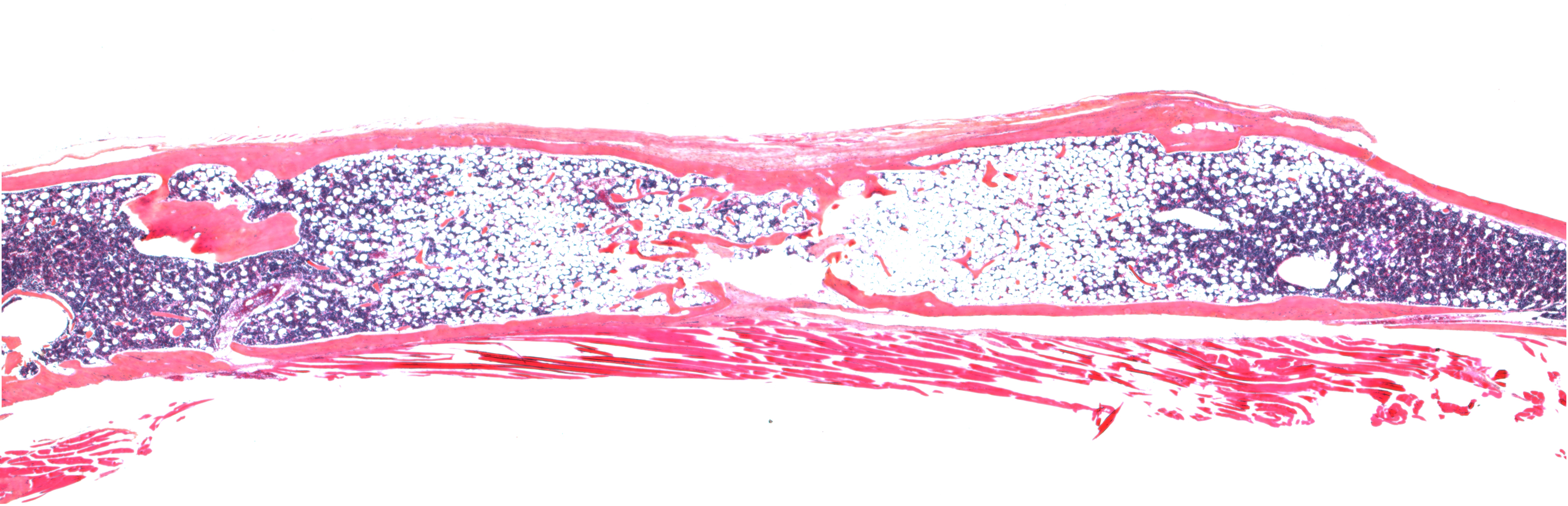

Supplement: S1 File — (ZIP) [file pone.0280634.s002.zip › Yamaguchi University/Figure 5/HE staining/Wild-type 12w(2).jpg]

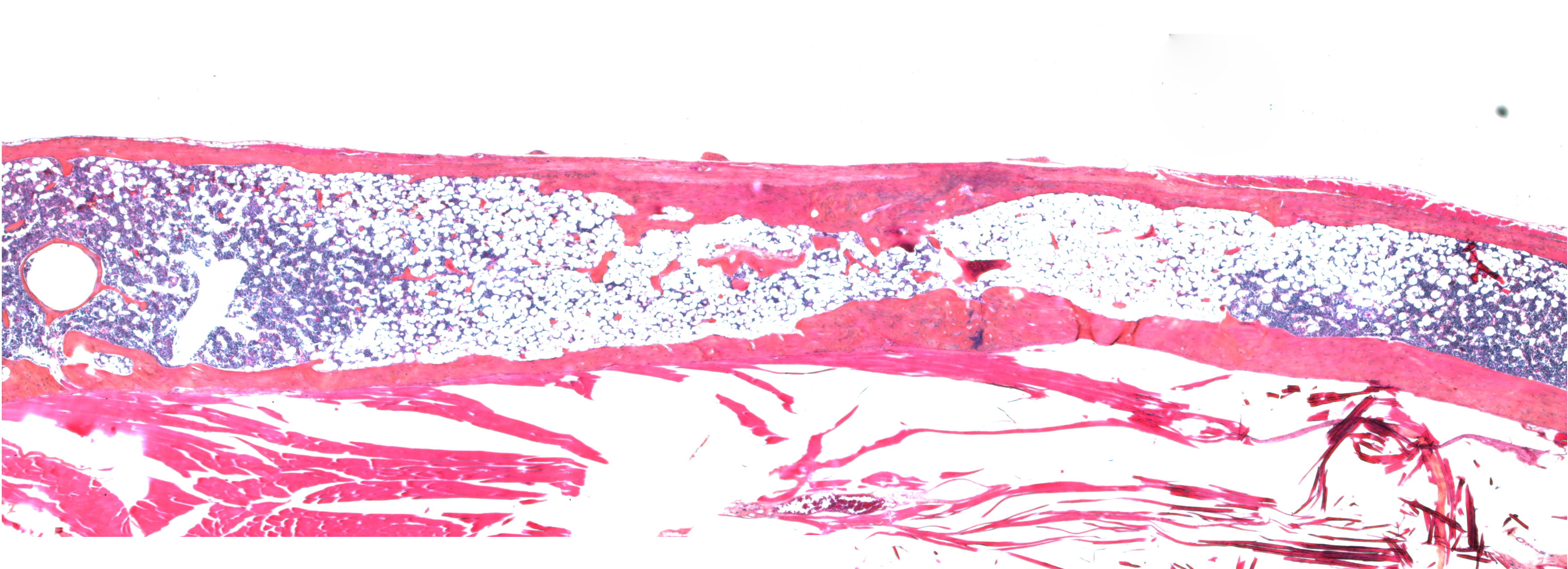

Supplement: S1 File — (ZIP) [file pone.0280634.s002.zip › Yamaguchi University/Figure 5/HE staining/Wild-type 12w(3).jpg]

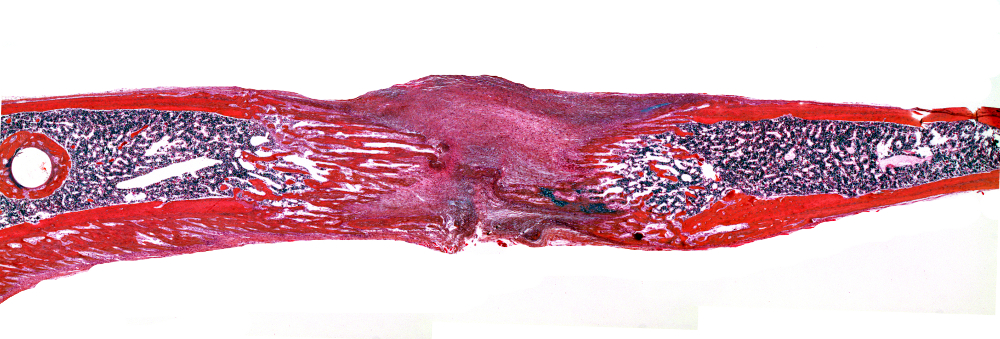

Supplement: S1 File — (ZIP) [file pone.0280634.s002.zip › Yamaguchi University/Figure 5/HE staining/Wild-type 3w.jpg]

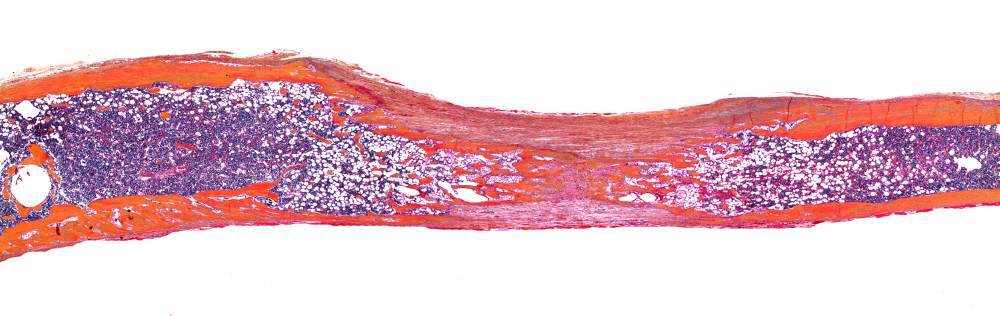

Supplement: S1 File — (ZIP) [file pone.0280634.s002.zip › Yamaguchi University/Figure 5/HE staining/Wild-type 4w.jpg]

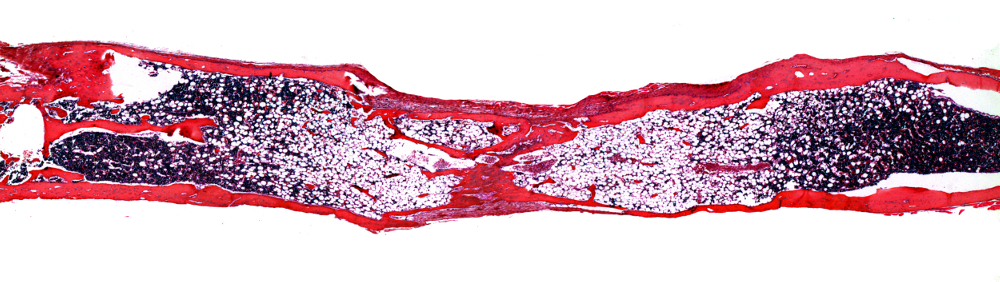

Supplement: S1 File — (ZIP) [file pone.0280634.s002.zip › Yamaguchi University/Figure 5/HE staining/Wild-type 6w.jpg]

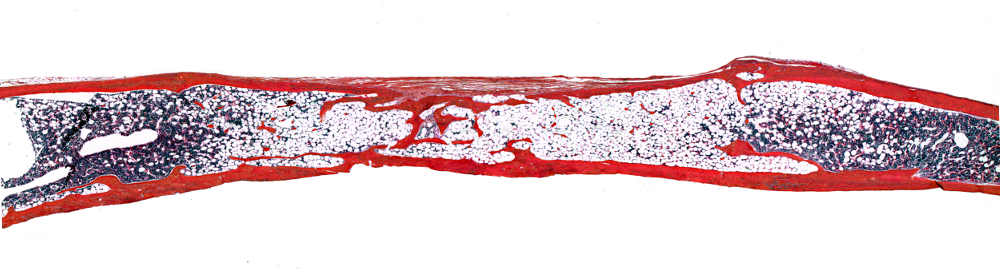

Supplement: S1 File — (ZIP) [file pone.0280634.s002.zip › Yamaguchi University/Figure 5/HE staining/Wild-type 8w.jpg]
