# Supplementary material for: Chondromodulin is necessary for cartilage callus distraction in mice
Source: PLoS One. 2023 Feb 16;18(2):e0280634. doi: 10.1371/journal.pone.0280634 (PMC9934371; doi:10.1371/journal.pone.0280634)
Supplement: Attachment — Submitted filename: PLOS response.docx [file pone.0280634.s003.docx]

Re: Manuscript ID: PONE-D-21-40664

Title: Chondromodulin is Necessary for Cartilage elongation and Complete Distraction Osteogenesis in Mice

We thank you and the reviewers for the very useful comments and your time for critical and careful review of our manuscript.

We have responded in a point-by-point fashion to the comments from the Editor and Reviewers and indicated how the suggestions have been incorporated in the revised manuscript where appropriate. We have clearly highlighted all the changes in the manuscript for easy identification. We believe the manuscript has been significantly improved and hope that it is suitable for publication in **PLOS ONE.**

We look forward to hearing from you at your earliest convenience.

Yours Sincerely,

Kiminori Yukata, MD, PhD.

(On Behalf of All Co-authors)

**Dear editors,**

We modified our manuscript according to PLOS ONE's style requirement. And, we added some information followed by editor's request.

**Responses to Reviewers’ Comments:**

Reviewer #1: This paper aims to demonstrate that chondromodulin is necessary for cartilage callus elongation during distraction osteogenesis in mice. They applied the same distraction protocol to mice without chondromodulin (cnmd null group) and a control group. Radiographies, immunohistochemical analysis and other results reveals that a poor callus and less production of cartilage at the cnmd null group. Therefore, authors suggest that regulates chondromodulin mechanical stress induced cartilage callus formation that is necessary for successful bone lengthening.
The publication of this work could help to understand the impact of chondromodulin on chondrogenesis and the difference in cartilage properties with and without chondromodulin. However, the present manuscript needs major changes because the authors obviate that there is controversy about the type of ossification during osteogenic distraction. Studies can be found in which ossification is mainly endochondral, intramembranous or both. They should be cited. Many studies have reported that ossification during distraction osteogenesis is primarily intramembranous, especially in large animals and models closer to humans. This should be addressed in the introduction and discussion of the article, and taken into account as a limitation of the article.

Response: Thank you very much for your critical review.　As reviewer suggested, in some situations (e.g., larger animals or shorter latency periods), bone lengthening occurs by predominantly intramembranous bone formation. Cartilage callus elongation and endochondral ossification may not be important, and, chondromodulin may not be necessary in such cases.　About this, we have added some sentences as a limitation.

Please see Page 15, Line 307-311.

Taking the above into account implies changing the focus of the manuscript on many points. For example, if there is no cartilage in the distraction callus, chondromodulin will not be necessary. In the title may be read “Chondromodulin is Necessary for … and Complete Distraction”. This would not be true in these cases, so it can confuse the reader. In my opinion, according to the outcomes, chondromodulin is being shown to be necessary to create cartilage distraction callus. However, I do not see it essential for carrying out the distraction, since in many cases the distraction is carried out by means of intramembranous ossification.

Response: Thank you very much for your good suggestion.　 We changed the title to “Chondromodulin is necessary for cartilage callus elongation during distraction osteogenesis in mice”

Please see the new Title in the revised manuscript and cover letter.

Another example, lines 271 to 273: “Cnmd null mutation resulted in the suppression of cartilage callus formation in distraction osteogenesis, and the newly generated tissue was fibrous”. This occurs in other studies with normal animals. Why? Discuss it in the article.
Response: Thank you very much for your good question.　Tissues after failed articular cartilage repair procedure are usually composed of fibrous connective tissues and fibrocartilage, which could lack chondromodulin expression. So, we added some sentences about it in the discussion section. Please see Page 13, Line 265-269.

Other comments:
Lines 162 to 175, in the text, parenthesis structures are used within parentheses, which makes reading extremely difficult. This section must be organized in another way to be understandable. It is advisable to use tables, footnotes or other resources.

Response: Thank you very much for your suggestion.　 We added the supplemental Table 1 about PCR primers’ information.

Lines 85 to 101 ¿How many animals were used for each sacrifice time point?
It is not clear how many time points and how many animals were used for each type of analysis. A table summarizing this information would be convenient at the beginning of the results section or end of M&M section.

Response: Thank you very much for your suggestion.

We described the number of mice in detail, which were used in this study.

Please see Page 5 line 87-96.

“Radiographs were taken weekly with a soft radiograph apparatus (CMB-2, SOFTEX, Japan) under anesthesia (n=10 for each WT and *Cnmd*^-/-^). Radiographic assessment of bone union was performed using postoperative radiographs at 8 weeks after osteotomy. Bone union was defined as when two of two cortices were bridged, partial union was defined as when either side of the cortical bone was not bridged, and non-union was defined as when both two cortices were not bridged. Mice were euthanized with isoflurane and carbon dioxide. For histology, mice were sacrificed 1, 2, 3, 4, 6, 8, and 12 weeks after osteotomy (n (n=4 for each genotype). For RNA extraction, mice were also sacrificed at 0, 1, 2, 3, 4, 5, 6, and 8 weeks after osteotomy (n=4 for each genotype).”
