# Supplementary material for: Chondromodulin is necessary for cartilage callus distraction in mice
Source: PLoS One. 2023 Feb 16;18(2):e0280634. doi: 10.1371/journal.pone.0280634 (PMC9934371; doi:10.1371/journal.pone.0280634)
Supplement: Attachment — Submitted filename: PLOS response 2.docx [file pone.0280634.s004.docx]

Re: Manuscript ID: PONE-D-21-40664R1

Title: Chondromodulin is Necessary for Cartilage elongation and Complete Distraction Osteogenesis in Mice

We thank you and the reviewers for the very useful comments and your time for critical and careful review of our manuscript.

We have responded in a point-by-point fashion to the comments from the Editor and Reviewers and indicated how the suggestions have been incorporated in the revised manuscript where appropriate. We have clearly highlighted all the changes in the manuscript for easy identification. We believe the manuscript has been significantly improved and hope that it is suitable for publication in **PLOS ONE.**

We look forward to hearing from you at your earliest convenience.

Yours Sincerely,

Kiminori Yukata, MD, PhD.

(On Behalf of All Co-authors)

**Dear editors,**

We added some information followed by editor's request.

**Responses to Reviewers’ Comments:**

Reviewer #2: Authors perform an in vivo analysis in a mice model to determine the role of chondromodulin in distraction osteogenesis. They do a careful study on the different tissues and their compositions in the distracted callus (histological, radiographic, inmunohistochemical studies). They found that chondromodulin inhibition reduce cartilage in the distracted callus.

The topic of the paper is interesting; authors recognise in the title that the conclusions are limited to the animal model they use (a mice model). In fact, there is much debate in literature about the possibility of a soft/cartilage callus under tensile load as in distraction osteogenesis. Authors state in the paper that the cartilage appears in small animals under this conditions, however they should discuss a little bit about the real mechanical environment in the mice tibia. In fact, I have some doubts about their external fixator capacity to isolate just tensile load in the callus while distracting, probably the compressive and bending loads are also important during the gait cycle. This aspect should be further discussed and the authors should try to determine the real mechanical environment in the callus.
Response: Thank you very much for your comment.

In a rabbit tibial lengthening model, Waanders et al. showed that the gap tissue was exposed not only to tension force but also to cyclic strain of about 15% of the tension force during walking [40].

It means that not only tensile load but also various mechanical forces affect the distraction segment.

So, we added some sentences in the discussion section (Page 15, Line 315-320).

Comments:
Lines 83-84 “The fibula was not broken” In figure 1 it seems that the fibula resulted broken due to the distraction itself, in time points 2-6 weeks after fracture. If this is not the case, the load sharing mechanism between tibia-fibula-fixator would be altered, please discuss a Little bit in the discussion section about the role of fibula.

Response: Thank you very much for your critical comment. As the reviewer pointed out, we did not break the fibula, but it was naturally broken and lengthened during the distraction period. (Page5, Line 83-84) So, we believe that the mechanical influence of fibula on tibial lengthening is small.

Lines 85-87. The distraction protocol is described, please include also the total distracted length to make the manuscript easier to read (it is already included in figure 1 legend).

Response: We added the total distracted length in the materials and method section. (Page 5, Lines 87-88)

Line 39 Typo “osteogenesis”

Response: We exchanged the word ‘chondrogeneses’ to ‘osteogenesis.’ (Page 3, Line 39)

Line 131 “in vitro” it should be in cursive fonts.

Response: Thank you very much. We corrected it. (Page 7, Line 132)

Figure 1. To use a nomenclature similar to the state of the art replace “Lag phase” by “Latency hase”

Response: Thank you very much for your suggestion. We replaced by ‘Latency phase’ throughout the manuscript and figures. (Line 85, 86, and Figure 2)

Figure 1. Include a scale bar in the radiograph

Response: We added a scale bar in the radiographs of figure 2. Also, we described about it in the figure legends.

Lines 160, 243, 251… Acronym of Vascular Endothelial Growth Factor should be better rewritten with capital letters VEGF. Same comment for Matrix metallopeptidase (MMP).

Response: Thank you very much for your critical review. We changed it as the reviewer suggested. Please see the corrected words marked in yellow.

Lines 309-313. One of the critical point of the study is cartilage formation in a distracted callus in contrast to previous studies in which intramembranous ossification occur under distraction “perhaps because the latency period was rather long”, the latency period is long for a mice model but other factors could also favour endochondral ossification for example the external fixator as described in the paper could produce some bending and compression movement during the gait of the anima. Please, comment about it also.

Response: Thank you very much for your comment.

In a rabbit tibial lengthening model, Waanders et al. showed that the gap tissue was exposed not only to tension force but also to cyclic strain of about 15% of the tension force during walking [40].

So, we believe that some bending and compression movement in addition to tension stress would affect cartilage callus formation.
