# Supplementary material for: Chondromodulin is necessary for cartilage callus distraction in mice
Source: PLoS One. 2023 Feb 16;18(2):e0280634. doi: 10.1371/journal.pone.0280634 (PMC9934371; doi:10.1371/journal.pone.0280634)
Supplement: Attachment — Submitted filename: PLOS response 3.docx [file pone.0280634.s005.docx]

Title: Chondromodulin is necessary for cartilage callus distraction in mice

We thank you and the reviewers for the very useful comments and your time for critical and careful review of our manuscript.

We have responded in a point-by-point fashion to the comments from the Editor and Reviewers and indicated how the suggestions have been incorporated in the revised manuscript where appropriate. We have clearly highlighted all the changes in the manuscript for easy identification. We believe the manuscript has been significantly improved and hope that it is suitable for publication in **PLOS ONE.**

We look forward to hearing from you at your earliest convenience.

Yours Sincerely,

Kiminori Yukata, MD, PhD.

(On Behalf of All Co-authors)

**Responses to Reviewers’ Comments:**

Reviewer #2: In general, I find authors have answer to my previous questions but they should justify their answers and more important they should include some discussion in the paper. Also, please highlight the modified parts in the manuscript.

In my previous review I commented “Lines 83-84 “The fibula was not broken” In figure 1 it seems that the fibula resulted broken due to the distraction itself, in time points 2-6 weeks after fracture. If this is not the case, the load sharing mechanism between tibia-fibula-fixator would be altered, please discuss a Little bit in the discussion section about the role of fibula.” And authors answered “Response: Thank you very much for your critical comment. As the reviewer pointed out, we did not break the fibula, but it was naturally broken and lengthened during the distraction period. (Page5, Line 83-84) So, we believe that the mechanical influence of fibula on tibial lengthening is small”
They recognize in the paper that the fibula was broken during distraction, but they do not include any discussion about this in the discussion section, I think the mechanical environment in the fracture site is modified due to the broken fibula the authors just answer to me “we believe that the mechanical influence of fibula on tibial lengthening is small”, please justify and include some comments in the discussion section.

I also commented about the possible bending and compression movement in the gap, even though they recognize it should exit, nevertheless there is no discussion in the paper. They say
Lines 315-317 “Waanders et al. showed that the gap tissue was exposed not only to tension force but also to cyclic strain of about 15% of the tension force during walking” Please rephrase, what do you mean by cyclic strain (tension or compression), one should assume the tension force will result in tension strain. Please rephrase.

Response: Thank you very much for your careful review and pointing out.

The reviewer recommended us to discuss about the mechanical environment of the lengthened segment. That’s a great idea. So, we added one paragraph about the discussion on it from line 281 to 300. Actually, the extent to which the fibula mechanically affected the tibial lengthening is unclear, but it definitely affected. So we mentioned it in the discussion section.

Typo:, line 106 “in situ” cursive.

Response: Thank you very much for your suggestion. We modified it in Line 106.

Finally, we changed the title of this manuscript to ‘Chondromodulin is necessary for cartilage callus distraction in mice.’

Also, we changed some words, which were highlighted yellow.

Because ‘callus distraction’ is more common than ‘callus elongation’
